# LRRK2 and RAB7L1 coordinately regulate axonal morphology and lysosome integrity in diverse cellular contexts

**DOI:** 10.1038/srep29945

**Published:** 2016-07-18

**Authors:** Tomoki Kuwahara, Keiichi Inoue, Vivette D. D’Agati, Tetta Fujimoto, Tomoya Eguchi, Shamol Saha, Benjamin Wolozin, Takeshi Iwatsubo, Asa Abeliovich

**Affiliations:** 1 Departments of Pathology, Cell Biology and Neurology, and Taub Institute, Columbia University, New York, NY, 10032, USA; 2 Department of Neuropathology, Graduate School of Medicine, The University of Tokyo, Tokyo, 113-0033, Japan; 3 Department of Pathology and Cell Biology, College of Physicians and Surgeons, Columbia University, New York, NY, 10032, USA; 4 Department of Pharmacology and Experimental Therapeutics and Department of Neurology, Boston University School of Medicine, Boston, MA, 02118, USA

## Abstract

*Leucine-rich repeat kinase 2* (*LRRK2*) has been linked to several clinical disorders including Parkinson’s disease (PD), Crohn’s disease, and leprosy. Furthermore in rodents, LRRK2 deficiency or inhibition leads to lysosomal pathology in kidney and lung. Here we provide evidence that LRRK2 functions together with a second PD-associated gene, RAB7L1, within an evolutionarily conserved genetic module in diverse cellular contexts. In *C. elegans* neurons, orthologues of LRRK2 and RAB7L1 act coordinately in an ordered genetic pathway to regulate axonal elongation. Further genetic studies implicated the AP-3 complex, which is a known regulator of axonal morphology as well as of intracellular protein trafficking to the lysosome compartment, as a physiological downstream effector of LRRK2 and RAB7L1. Additional cell-based studies implicated LRRK2 in the AP-3 complex-related intracellular trafficking of lysosomal membrane proteins. In mice, deficiency of either RAB7L1 or LRRK2 leads to prominent age-associated lysosomal defects in kidney proximal tubule cells, in the absence of frank CNS pathology. We hypothesize that defects in this evolutionarily conserved genetic pathway underlie the diverse pathologies associated with LRRK2 in humans and in animal models.

The *LRRK2* gene encodes a large cytosolic protein that includes kinase, GTPase, and multiple protein interaction domains. Rare mutations in *LRRK2* lead to familial autosomal dominant forms of Parkinson’s disease (PD)^1^,^2^. In addition, common genetic variants at the *LRRK2* locus have been associated with non-familial PD risk in several large genome wide association studies (GWAS)^3^,^4^. More recently, GWAS have also linked common variants at the *LRRK2* locus with Crohn’s disease, inflammatory bowel disease (IBD), and leprosy^5^,^6^,^7^, and mutations in *LRRK2* have been described in cancers^8^,^9^. These conditions span a remarkably broad range of organ systems.

Human genetic studies of PD patient cohorts have previously suggested that common variants at LRRK2 function coordinately with variants at a second PD risk-associated locus, *PARK16*, to increase PD risk, as human genetic variants at these 2 loci impact PD risk non-additively^10^,^11^. The *PARK16* locus encompasses several genes including *RAB7-like variant 1* (*RAB7L1*), which is a member of an evolutionarily conserved subset of the Rab family of G proteins, consisting of RAB7L1, RAB32 and RAB38. These Rab proteins have been specifically implicated in endosome-lysosome protein sorting^12^,^13^. A *C. elegans* orthologue of RAB7L1 as well as of RAB32 and RAB38, termed *gut granules loss* (*glo-1*), is essential for the biogenesis of lysosome-related organelles, termed gut granules, in intestinal cells^14^. *glo-1* also regulates axon morphology in a subset of *C. elegans* neurons^15^.

LRRK2 is a large, cytosolic protein composed of 2527 amino acids harboring multiple characteristic protein domains. Like RAB7L1, LRRK2 has previously been implicated in neurite morphology and lysosomal integrity^16^,^17^,^18^,^19^. Furthermore, LRRK2 physically interacts with RAB7L1^10^,^20^. However, the mechanism by which these proteins may coordinately regulate neurites and lysosomes, *in vitro* or *in vivo*, remains unclear. Rodents deficient in *Lrrk2* harbor age-dependent, prominent lysosomal defects in renal proximal tubule cells^21^,^22^,^23^,^24^ and in lung type II pneumocytes^25^ – two cell types that are known to have particularly active lysosomal transport compartments, further supporting a broad role for LRRK2 in lysosomal function. Surprisingly, such animals have been reported to display only limited and inconsistent brain pathology.

We herein initially pursued the physiological role of the LRRK2 orthologue LRK-1 in nematode *C. elegans* neurons. Genetic studies revealed that LRK-1 negatively regulates axonal elongation within mechanosensory neurons. Furthermore, LRK-1 functions within a genetic pathway consisting of GLO-1 as well the adaptor protein complex 3 (AP-3) component, APB-3, which is essential for vesicular endosomal trafficking to lysosomes or lysosome-related organelles (LROs)^26^,^27^. Further genetic epistasis analyses suggested a model where LRK-1 acts downstream of GLO-1 and upstream of APB-3. Mammalian cell-based *in vitro* assays supported a functional relationship between LRRK2 and AP-3 in the trafficking of lysosome-bound proteins. Finally in mice, we observed a striking similarity between the phenotypes of animals deficient in either RAB7L1 or LRRK2, with normal appearing midbrain dopaminergic neurons but prominent kidney pathology characterized by lysosomal inclusions in proximal tubule cells. Double mutant mice displayed a non-additive phenotype, consistent with a common mechanism of action. Taken together, these results implicate a physiological RAB7L1-LRRK2 pathway in the regulation of AP-3-dependent functions, such as intracellular trafficking of lysosome-bound proteins and the regulation of axonal morphology, in diverse cellular and organismal contexts.

## Results

### LRK-1/LRRK2 regulates axon termination in *C. elegans*

**Figure 1:**
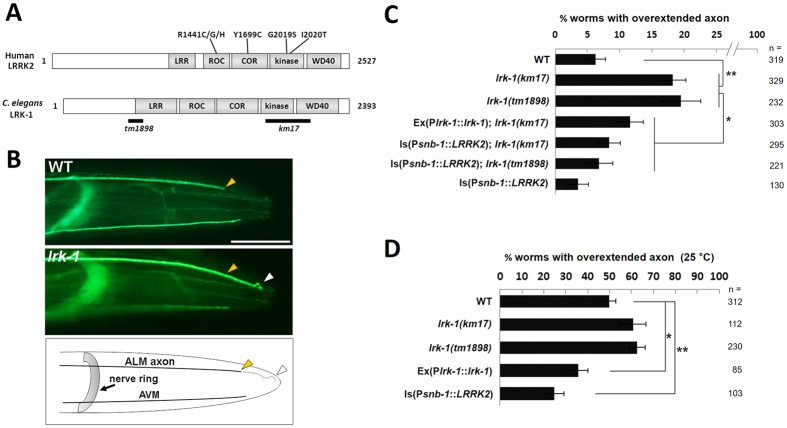
LRK-1/LRRK2 regulates axon termination in *C. elegans* neurons. **(A)** Schematic of primary structures of human LRRK2 and *C. elegans* LRK-1 proteins, as labelled. Positions of PD-associated familial missense mutations in LRRK2, and of deletion alleles in LRK-1, are shown. **(B)** Axonal processes of *C. elegans* mechanosensory neurons (ALM and AVM) were marked by selective expression of GFP using the *mec-7* promoter (P*mec-7*::GFP) and visualized by fluorescence microscopy. ALM neurons extend a single axon that normally terminates at a distance from the tip of the nose (yellow arrowhead) in WT worms. However, in *lrk-1* mutants, ALM axon frequently overextends to the tip of the nose and subsequently reverses course, resulting in a hook-like structure (white arrowhead). Schematic drawing of the ALM and AVM axon structures is shown at the bottom; anterior is to the right; scale bar = 50 μm. **(C)** Two independent *lrk-1* mutant strains (*km17* and *tm1898*) cultured at 20 °C showed overextension of ALM axons. This was rescued by transgenic expression of LRK-1 under its own promoter (P*lrk-1::lrk-1*) or of human wild-type LRRK2 (P*snb-1::LRRK2*) under the synaptobrevin promoter. Percentages exhibiting overextension of ALM axons are shown. Is: chromosomally integrated strain, ex: extrachromomally overexpressing strain. Data represent mean ± SEM, *p < 0.05, **p < 0.01; Samples numbers n are listed to the right of bar graphs. **(D)** Overextension of ALM axons was more frequently observed in nematodes cultured at 25 °C than at 20 °C, and this temperature-sensitive overextension was rescued by transgenic expression of LRK-1 or human LRRK2. Data represent mean ± SEM, *p < 0.05, **p < 0.01.

To investigate the *in vivo* role of LRRK2 in a genetically tractable model organism, we initially turned to the nematode *C. elegans*. *C. elegans* has been used extensively as a model to describe mechanisms of action of neurodegenerative disease genes^28^,^29^, and an orthologue of mammalian LRRK2, termed LRK-1, have been described in *C. elegans*^30^ (Fig. 1A). Because previous studies implicated LRRK2 in the regulation of mammalian neurite morphology^16^,^18^, we initially examined axonal morphology parameters in nematodes with mutations in *lrk-1*, focusing on the well described ALM mechanosensory neuron axons. We analyzed nematodes that harbor either of two independent *lrk-1* mutant alleles: *km17*, a deletion lacking the C-terminal portion of the gene that includes the kinase domain, or *tm1898*, a frameshift mutation within the leucine-rich repeat region that is predicted to result in a truncated protein lacking all known functional domains (Fig. 1A). *lrk-1* mutants exhibited a significantly increased frequency of ALM axon overextension: thus although ALM neurons typically extend a single axon anteriorly which terminates caudal to the tip of the nose, *lrk-1* mutants occasionally harbored ALM axons that extended all the way to the tip of the nose and then folded back, resulting in a hook-like structure (Fig. 1B). Quantitative analyses revealed that both the *km17* and *tm1898 lrk-1* mutant strains exhibited an increased frequency of ALM axon overextension, as quantified by fluorescent microscopy of live nematodes (relative to wild-type; Fig. 1B,C). This axonal overextension phenotype was rescued by transgenic expression of *C. elegans* LRK-1 driven by its own promoter (Fig. 1C). ALM axon overextension was similarly rescued by expression of human wild-type LRRK2 driven by the neuron-specific *snb-1* promoter (Fig. 1C), suggesting that the role of LRK-1/LRRK2 on axon termination is conserved across species and that the gene acts cell-autonomously within the nervous system.

As axonal overextension phenotypes in *C. elegans* are known to be temperature-dependent^15^, we also examined animals cultured at a higher temperature (25 °C) throughout their lifetime. About 50% of wild-type nematodes showed overextension of ALM axons at 25 °C, and overexpression of LRK-1 or human LRRK2 led to a significant reduction in axon extension compared with wild-type worms (Fig. 1D). We further examined animals that overexpress PD-associated mutant *LRRK2-G2019S* or *LRRK2-R1441C*. Overextension of ALM axons at 25 °C was similarly suppressed by expression of either *LRRK2-G2019S* or *LRRK2-R1441C*, as observed in animals expressing wild-type *LRRK2*; the effect of PD-associated mutations was not evident in this axon phenotype (Supplementary Fig. S1). Together, these data support the notion that LRK-1/LRRK2 physiologically regulates axonal morphology in *C. elegans* mechanosensory neurons.

### LRK-1/LRRK2 function in a GLO-1 pathway regulating ALM axon termination

**Figure 2:**
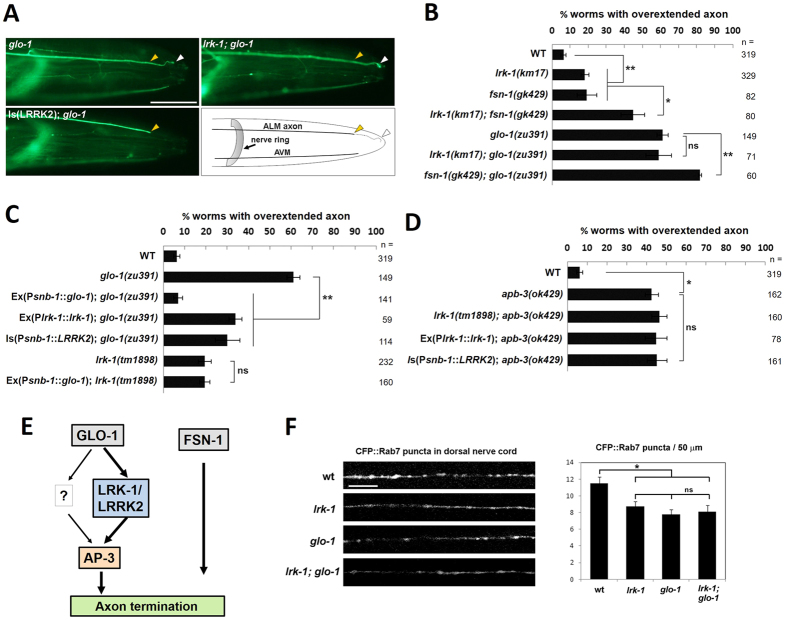
LRK-1/LRRK2 function within GLO-1-AP-3 pathway for axon termination. **(A)** Visualization of ALM axonal processes expressing GFP in the indicated strains, as shown in Fig. 1B. The normal termination site (yellow arrowhead) and the overextension site (white arrowhead) are indicated. Schematic drawing is shown at the bottom right; anterior is to the right; scale bar = 50 μm. **(B)** Analysis of ALM axon termination phenotypes in single- and double- mutant nematodes suggests that LRK-1 acts in a common pathway with GLO-1, but not with FSN-1. Percentages exhibiting overextension of ALM axons are shown for each strain. Worms were cultured at 20 °C. Data represent mean ± SEM, *p < 0.05, **p < 0.01, ns: not significant. **(C)** In transgenic rescue experiments, nematode LRK-1 or human LRRK2 overexpression rescued *glo-1* mutation-associated axon overextension, whereas GLO-1 overexpression failed to rescue *lrk-1* mutation. Percentages exhibiting overextension of ALM axons are shown for each strain. Is: chromosomally integrated strain, ex: extrachromomally overexpressing strain. Worms were cultured at 20 °C. Data represent mean ± SEM, **p < 0.01, ns: not significant. **(D)** *apb-3* mutants exhibit the same ALM axon overextension phenotype observed in *lrk-1* mutants, whereas double mutants in both genes are equivalent to the single mutants (non-additive). Percentages of indicated strains showing overextension of ALM axons at 20 °C are shown. Data represent mean ± SEM, *p < 0.01, ns: not significant. **(E)** A schematic of the genetic pathways regulating ALM axon termination. LRK-1 appears to act downstream of GLO-1, upstream of AP-3 and in parallel with FSN-1. There might be LRK-1-dependent and independent pathways in the course from GLO-1 to AP-3. **(F)** Left: Rab7-CFP positive puncta, typically localized to late endosomes and lysosomes, were visualized by fluorescent microscopy along the dorsal nerve cord of the indicated mutant strains harboring the transgenic marker *juEx989* (P*unc-25*::*cfp*::*rab-7*). Representative images are shown; Scale bar = 10 μm. Right: quantitative analysis of the number of CFP::Rab7 puncta in a 50 μm region along the dorsal nerve cord of the indicated strains. Puncta numbers were significantly reduced in *lrk-1*, *glo-1* and *lrk-1;glo-1* mutants. Data represent mean ± SEM, n = 15, *p < 0.01, ns: not significant.

The overextension defects in ALM axons seen in the context of LRK-1 deficiency closely resembled the phenotypes previously described in the context of mutations in *glo-1*, an orthologue of *RAB7L1*, as well as the components of AP-3 complex, an endo-lysosomal clathrin adaptor complex that acts downstream of GLO-1^15^. The GLO-1-AP-3 genetic pathway has also been identified as responsible for the biogenesis of gut granules, a lysosome-related organelle in the *C. elegans* intestinal tract^14^,^31^. We first investigated the genetic relationship between *lrk-1* and *glo-1*. We confirmed that over 50% of *glo-1*(*zu391*) mutants showed an ALM axonal overextension phenotype, as previously reported^15^ (Fig. 2A,B). Furthermore, *lrk-1*(*km17*); *glo-1*(*zu391*) double-mutant animals harbored an axonal overextension phenotype that appeared equivalent to the phenotype of single mutant animals (non-additive, Fig. 2A,B). This suggested that LRK-1 functions within a common pathway with GLO-1.

A previous study reported that axon termination in ALM neuron was regulated not only by the GLO-1 pathway but also by a parallel pathway mediated by FSN-1, an F-box synaptic protein that functions within a Skp/Cullin/F-box (SCF) protein complex^15^. The analysis of single- and double- mutant animals harboring *glo-1(zu391)* or *fsn-1(gk429)* alleles, that are predicted to be functionally null^14^, revealed that GLO-1 and FSN-1 appear to act in separate, parallel pathways, rather than within a common linear pathway (Fig. 2B); the percentage of nematodes showing ALM axon overextension was significantly higher in *lrk-1(km17); fsn-1(gk429)* double mutants than in *fsn-1(gk429)* or *lrk-1(km17)* single mutants. This result was consistent with these genes functioning in parallel (additive) pathways, and further support a model where LRK-1 acts in a GLO-1 genetic pathway (Fig. 2E).

### LRK-1/LRRK2 acts downstream of GLO-1 in the regulation of axon termination

To investigate the epistatic relationship of *lrk-1* and GLO-1, we performed transgenic rescue experiments. In *glo-1* mutant animals, transgenic expression of either *C. elegans* GLO-1 or LRK-1, or of human LRRK2, rescued the axon overextension phenotype of ALM neurons (Fig. 2C), although rescue by *C. elegans* LRK-1 or human LRRK2 was incomplete, in contrast to rescue by GLO-1 transgenic expression. In *lrk-1* mutant *C. elegans*, expression of GLO-1 failed to rescue the axonal overextension phenotype (Fig. 2C), whereas expression of either LRK-1 or human LRRK2 was effective (Fig. 1C). These data suggested that LRK-1 functions downstream of GLO-1 in a linear pathway that regulates axonal morphology at ALM neurons (Fig. 2D).

### AP-3 as a candidate effector of LRK-1 and GLO-1 in the regulation of axon termination

Prior studies have implicated the AP-3 complex, which is required for vesicular endosomal trafficking to lysosomes or lysosome-related organelles (LROs)^26^,^27^ in neurons and other cells, as a putative downstream effector of GLO-1 in the regulation of axonal morphology at ALM neurons. Specifically *apb-3*, which encodes the beta subunit of the AP-3 complex, has been reported to play an essential role downstream of *glo-1* in the context of axonal overgrowth^15^, as well as in the biogenesis of lysosome-like structures^14^,^31^. We thus next investigated the genetic relationship between LRK-1/LRRK2 and the AP-3 component *apb-3*. Mutant nematodes harboring a null allele in *apb-3* (*apb-3(ok429)*) displayed ALM axon overextension at 20 °C, akin to *lrk-1* and *glo-1* mutants (Fig. 2D), as expected. Double mutants deficient in both *lrk-1* and *apb-3* did not show further enhancement of axon overextension compared with each single mutant (Fig. 2D), suggesting that LRK-1 and APB-3 may act in a common genetic pathway. Furthermore, transgenic expression of *C. elegans* LRK-1 or human LRRK2 did not rescue the axon overextension of *apb-3* mutants (Fig. 2D), in contrast to the effective rescue of *glo-1* mutants or *lrk-1* mutants by the *C. elegans* LRK-1 or human LRRK2 transgenes (Figs 1C and 2C). These results, taken together with prior analyses^15^, support a model in which LRK-1 functions downstream of GLO-1 and upstream of AP-3 to modify axonal termination (Fig. 2E).

The penetrance of the axonal elongation phenotype in *lrk-1* mutants (~20%) appears significantly lower than the penetrance seen in the *glo-1* or *apb-3* mutants (40–60%). We thus hypothesized that within the GLO-1-LRK-1-AP-3 pathway, LRK-1 may play a partially redundant role (Fig. 2E). To further pursue this pathway, we also investigated the role of LRK-1 in a second phenotype associated with both GLO-1 and AP-3 – the accumulation of autofluorescent gut granules, which are lysosome-related organelles, in intestinal cells^14^. In this context, *lrk-1* mutants appeared unaltered relative to wild type controls, whereas *glo-1* or *apb-3* mutants completely lack gut granules, as previously reported (Supplementary Fig. S2A). Thus, in some contexts GLO-1 and AP-3 appear to function independently of LRK-1.

### LRK-1 and GLO-1 modulate endo-lysosomal accumulation in motor neuron axons

Prior studies suggest that in neurons, the GLO-1-AP-3 pathway not only regulates axonal elongation, but additionally participates in axonal endo-lysosomal trafficking or maturation. Thus loss of GLO-4, which encodes an essential GTP exchange factor for GLO-1 in this pathway, has been reported to result in the defective accumulation of an endo-lysosome marker, cyan fluorescent protein (CFP)-Rab7, along a subset of motor neuron axons^15^. Consistent with this, quantitative analysis of CFP-Rab7 fluorescent puncta in the dorsal nerve cord revealed a significant reduction in axonal puncta number in either *glo-1* or *lrk-1* mutant animals, compared with wild-type animals (Fig. 2F). Puncta number in *lrk-1;glo-1* double mutants was not further decreased compared with that in *lrk-1* mutants or *glo-1* mutants (Fig. 2F), supporting the notion that LRK-1 and GLO-1 act in a common genetic pathway. The reduction of CFP-Rab7 puncta did not appear to be due to a general defect in axonal structure in motor neurons, as the number of puncta marked by the transgenic presynaptic marker, synaptobrevin (SNB-1)-mCherry, along the dorsal nerve cord was not decreased in *lrk-1*, *glo-1*, or *lrk-1;glo-1* double mutants (Supplementary Fig. S2B). These results support a role for LRK-1 and GLO-1 in a common genetic pathway that regulates endo-lysosomal trafficking or maturation.

### LRRK2 interacts with AP-3 to regulate intracellular trafficking of lysosomal-targeted membrane proteins

**Figure 3:**
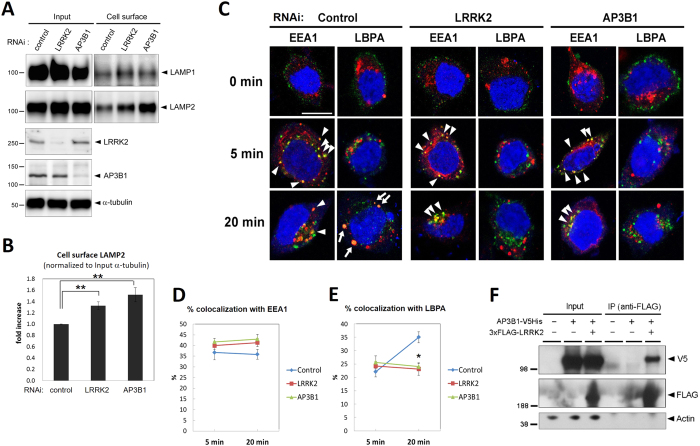
LRRK2 interacts with AP-3 to regulate intracellular recycling of lysosomal membrane proteins. **(A)** Cell-surface biotinylation analysis of HEK293 cells treated with LRRK2, AP3B1 or non-targeting siRNA as indicated. Representative western blot images are shown. **(B)** Quantification of cell surface accumulation of LAMP2 as shown in (**A**) from four independent experiments. Signals were normalized by the intensities of α-tubulin bands in input fractions. Data represent mean ± SEM, **p < 0.01. **(C)** Knockdown of LRRK2 as well as AP3B1 delays early-to-late endosomal trafficking of endocytosed LAMP1. N2a cells pretreated with either LRRK2, AP3B1 or non-targeting siRNA for 72hrs were incubated with Alexa488-labeled anti-LAMP1 antibody for 30 min at 4 °C, followed by incubation for 0, 5 or 20 min at 37 °C as indicated on the left. Cells were fixed and immunostained for the early endosomal marker EEA1 or the late endosomal marker LBPA as indicated (Red), and nuclei were stained with DRAQ5 (Blue). Colocalization of endocytosed LAMP1 (Green) with EEA1 or LBPA is depicted by arrowheads and arrows, respectively. Scale bar in upper left picture = 10 μm; all pictures are displayed at the same magnification. **(D,E)** Quantification of colocalization of Alexa488-labeled LAMP1 with EEA1 (**D**) and LBPA (**E**) at 5 min and 20 min after internalization. Data represent mean ± SEM; n ≧ 15 cells in three independent wells per group; *p < 0.01. **(F)** Immunoprecipitation (IP) was performed from lysates of SH-SY5Y cells transfected with 3xFLAG-tagged LRRK2 and V5-tagged AP3B1. IP with anti-FLAG antibody co-precipitated AP3B1. Arrowheads indicate the expected protein sizes. β-Actin is shown as a control.

We next sought to investigate the functional relationship between LRRK2 and the AP-3 complex, implicated by the above *C. elegans* genetic studies, using mammalian cultured cells. Prior studies have established an essential role for the AP-3 complex in the intracellular trafficking of major lysosomal membrane proteins (LMPs) such as LAMP1 and LAMP2^32^,^33^. In humans, mutations in the β3A subunit of the AP-3 complex, also termed as AP3B1, cause Hermansky-Pudlak Syndrome (HPS), a rare disorder characterized by defective lysosome-related organelles such as melanosomes, and altered lysosome appearance in various cell types^32^,^34^. In fibroblasts from HPS patients harboring mutations in *AP3B1* gene or from mice lacking AP-3 subunits such as AP3B1, LMPs accumulate abnormally at the cell surface plasma membrane, rather than at lysosomal membranes, possibly as a result of their missorting^32^,^33^. We thus examined whether LRRK2 has a similar role in the intracellular trafficking of LMPs. First, cell surface-localized LAMP1 or LAMP2 was examined using a cell-surface biotinylation assay in human embryonic kidney HEK293 cells treated with LRRK2 or AP3B1 siRNA. We observed significant accumulation of both LAMP1 and LAMP2 at the cell surface in cells treated with either LRRK2 siRNA or AP3B1 siRNA, compared with control siRNA-treated cells (Fig. 3A,B, Supplementary Fig. S3A), consistent with defective trafficking of LMP proteins. We additionally examined cell surface LAMP1/2 in cells expressing PD-associated LRRK2 mutants (G2019S, R1441C), but the pathogenic effects of them were not evident in this system (Supplementary Fig. S3B–S3D).

We next examined more directly whether LRRK2 influences the endosomal transport of cell surface LAMP1. Mouse neuroblastoma N2a cells pretreated with LRRK2, AP3B1, or non-targeting siRNA were surface-labeled with an Alexa488-conjugated antibody to the extracellular domain of LAMP1 for 30 min at 4 °C. To allow for internalization, the LAMP1 antibody-labeled cells were subsequently maintained at 37 °C for a period of time (0 min, 5 min, or 20 min), after which the cells were fixed and immunostained for early or late endosome marker (early endosome antigen-1 [EEA1] or lysobisphosphatidic acid [LBPA], respectively). In cells that were maintained at 4 °C, the LAMP1-antibody complex appeared restricted to the cell surface, whereas in cells placed for 5 or 20 min at 37°C, the complexes co-localized with endosome markers as well (Fig. 3C). Accumulation at early endosomes at these time points appeared similar among each siRNA-treated cells, whereas accumulation at late endosomes increased progressively in cells treated with control siRNA (Fig. 3D,E). In contrast, cells treated with siRNA for LRRK2 or AP3B1 failed to display a progressive increase in the accumulation of LAMP1-antibody complexes at late endosomes (Fig. 3E).

To further investigate the link between LRRK2 and the AP-3 complex in mammalian cells, we performed co-immunoprecipitation (IP) studies in transfected human neuroblastoma SH-SY5Y cells. IP of 3xFLAG-tagged human LRRK2 with an anti-FLAG antibody effectively co-precipitated V5-tagged human AP3B1, whereas AP3B1-V5 was not co-precipitated in the absence of 3xFLAG-LRRK2 (Fig. 3F). Furthermore, expression of LRRK2 stabilized co-expressed AP3B1, and expression of AP3B1 stabilized co-expressed LRRK2 in HEK293 cells, supporting intracellular co-association (Supplementary Fig. S3E–S3H). Taken together, these results support the notion that LRRK2 functions in AP-3-related intracellular recycling of LMPs.

### *Lrrk2* and *Rab7l1* deficient mice exhibit common lysosomal pathology in renal proximal tubule cells

**Figure 4:**
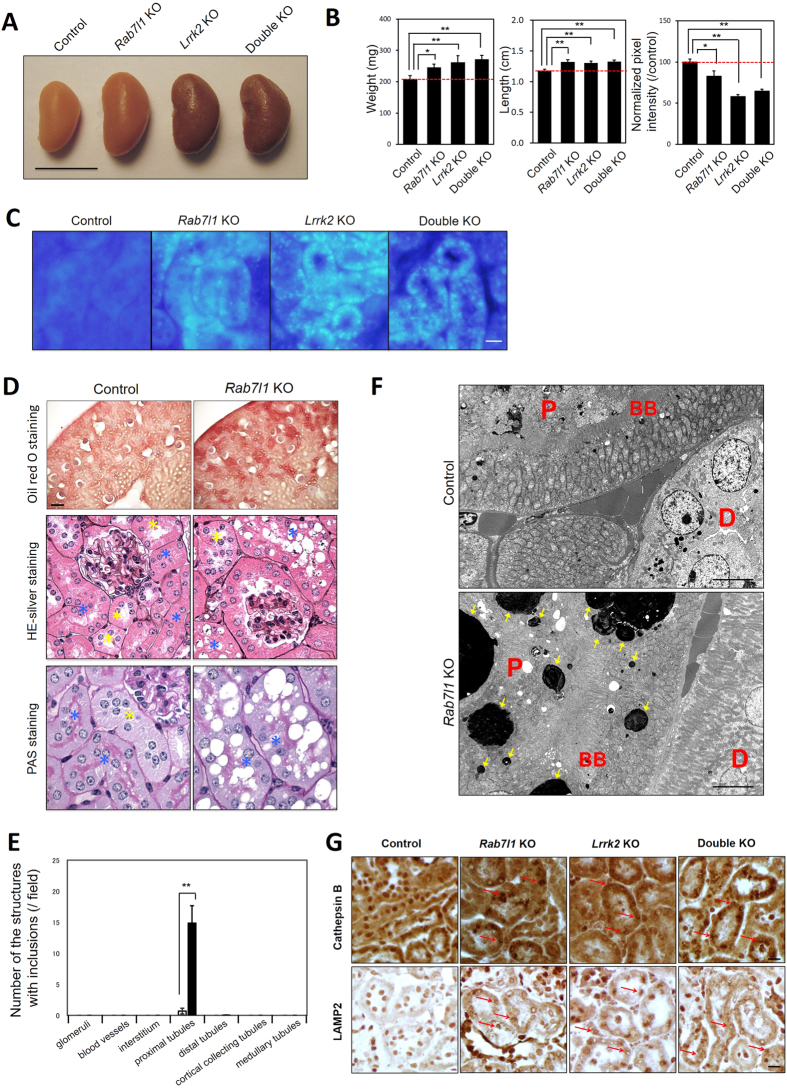
*Rab7l1* and *Lrrk2* deficient mice exhibit common lysosomal pathology in renal proximal tubule cells. **(A)** Gross appearance of representative kidneys from *Rab7l1* KO, *Lrrk2* KO, double KO or littermate control mice at 11–14 months of age. Scale bar = 10 mm. **(B)** Kidney weight and length were significantly greater in *Rab7l1* KO, *Lrrk2* KO or double KO mice compared with control mice as labeled. The color of kidney was significantly much darker in the mutants. Data represent mean ± SEM. n = 6 (control), 5 (*Rab7l1* KO), 5 (*Lrrk2* KO), 4 (double KO). *p < 0.05, **p < 0.01. **(C)** Auto-fluorescent granules were readily apparent in kidney proximal tubule cells of *Rab7l1* KO, *Lrrk2* KO or double KO mice at 11–14 months of age, but not in kidney tissue from control mice. Scale bar = 50 μm. **(D)** Histological analyses of kidney from *Rab7l1* KO mice. Oil red O staining (top row) revealed the accumulation of lipids in these vacuoles. Jones methenamine silver with eosin counterstaining (middle row) or PAS staining (bottom row) revealed the formation of extensive clear intracytoplasmic vacuoles in kidney proximal tubule cells of 10-month-old *Rab7l1* KO mice. Scale bar = 100 μm (Oil red O). Original magnifications: ×400 (HE-silver) and ×600 (PAS). **(E)** Quantification of the number of the structures with the intracytoplasmic vacuoles per 20× microscopic field. The counts in each indicated kidney tissue were made in at least 20 different 20× microscopic field per kidney. White bar: Control (*Rab7l1* (+/−)) mice; black bar: *Rab7l1* KO mice. Data represent mean ± SEM. n = 3 per genotype. **p < 0.01. **(F)** Electron microscopic analysis revealed the accumulation of electron-dense material (arrows) within enlarged secondary lysosomes in proximal tubule cells of 10-month-old *Rab7l1* KO mice. These inclusions are rounded, electron dense, of varying size and often have a whorled or lamellated texture (see Figure S3G). P, proximal tubule cells; D, distal tubule cells; BB, brush border. Scale bar = 5 μm. **(G)** Immunostaining of kidney sections revealed the accumulation of lysosomal proteins Cathepsin B and LAMP2 in 11- to 14-month-old *Rab7l1* KO, *Lrrk2* KO and double KO mice. Red arrows indicate the immunopositive signals. Scale bars = 20 μm.

To further relate the physiological role of LRRK2 with GLO-1/RAB7L1 in a higher-order organism, we investigated mutant mice deficient in *Lrrk2*, *Rab7l1*, or both. *Lrrk2* deficient mice (*Lrrk2*^*−/−*^; hereafter *Lrrk2* KO) exhibited significantly enlarged and grossly discolored kidneys at 11–14 months of age (Fig. 4A,B), as previously reported^21^,^22^,^23^,^24^. Analysis of *Rab7l1* homozygous mutant mice (*Rab7l1*^*−/−*^ ; hereafter *Rab7l1* KO), harboring a transgenic insertion-trap cassette between exons 3 and 4 (Supplementary Fig. S4A,S4B), revealed a very similar phenotype of renal enlargement and discoloration, as observed in *Lrrk2* KO mice (Fig. 4A,B), although the phenotype of the *Rab7l1* mutant mice appeared less severe. Double mutant mice homozygous for both *Rab7l1* and *Lrrk2* deficiency (hereafter double KO mice) exhibited renal enlargement that appeared non-additive, relative to single *Rab7l1* or *Lrrk2* deficiency alone (Fig. 4A,B), suggesting the two genes act in a common genetic pathway. Brain sections from *Lrrk2* KO, *Rab7l1* KO or double KO mice exhibited no evidence of midbrain dopamine neuron loss or striatal dopaminergic axon degeneration, as determined by tyrosine hydroxylase immunohistochemistry (Supplementary Fig. S4C,D).

Histological examination of kidney tissue sections from *Rab7l1* KO mice, *Lrrk2* KO mice or double KO mice at 11–14 months of age showed an accumulation of auto-fluorescent lipofuscin granules within proximal tubule cells of single and double KO mice (Fig. 4C). This phenotype was not observed in control littermate heterozygous mice at this age range (Supplementary Fig. S4E) nor in younger mutant animals (6–8 weeks old; Supplementary Fig. S4E). Oil red O staining of *Rab7l1* KO kidney confirmed extensive accumulation of lipids within lipofuscin granules (Fig. 4D). Additional histological analyses of *Rab7l1* KO kidney by Jones methenamine silver with eosin counterstain (HE-silver) and periodic acid-Schiff (PAS) staining revealed the accumulation of clear vacuoles highly selectively in renal proximal tubule cells (Fig. 4D); such pathology was almost never observed in other kidney structures including renal glomeruli, vessels, cortical distal tubules, cortical collecting tubules, and medullary tubules (Fig. 4E). Electron microscopic analyses demonstrated the accumulation of electron-dense intracytoplasmic material in the renal proximal tubules of *Rab7l1* KO mice (Fig. 4F, Supplementary Fig. S4F,G) consistent with engorged secondary lysosomes. These findings in *Rab7l1* mutant mice appear nearly identical to the renal pathology previously reported in other *Lrrk2* KO animals^22^,^24^.

We performed immunohistochemical (IHC) and immunoblot analyses of 10-month old mutant mice tissues. In kidneys of *Rab7l1* KO, *Lrrk2* KO, or double KO mice, the lysosomal marker proteins Cathepsin B and LAMP2 were increased in accumulation compared to control mice, as expected (Fig. 4G). LRRK2 protein levels were not altered in *Rab7l1* KO brain or kidney tissues (Supplementary Fig. S5A). We further examined the accumulation of AP-3 complex components, as well as of putative AP-3 complex-dependent cargo proteins, in these different mutant mouse strains. The levels of total AP-3 complex component AP3B1, or of phosphorylated AP3B1, did not appear altered in kidney or brains of *Lrrk2* KO or *Rab7l1* KO mice (Supplementary Fig. S5B,S5C). Two previously described AP-3 cargo proteins, LIMP2/SCARB2 and ZnT3, were significantly accumulated in renal tissues from *Lrrk2* KO mice but not in renal tissue from *Rab7l1* KO mice (Supplementary Fig. S5C). However, IHC analyses of kidney tissue revealed an altered subcellular localization of LIMP2, to enlarged secondary lysosome-like structures, in either *Rab7l1* KO or *Lrrk2* KO kidney tissue (Supplementary Fig. S5D). The relatively modest changes seen in the accumulation of AP-3 complex-associated cargo in *Rab7l1* KO mouse kidney tissue, relative to *Lrrk2* deficient tissue, is consistent with the milder histopathological findings observed in *Rab7l1* deficient mice.

## Discussion

We herein pursued physiological mechanisms of action of LRRK2 and RAB7L1 using *C. elegans*, mammalian cell culture, and mouse deficiency models. In *C. elegans* neurons, we identified a regulatory role for the *C. elegans* LRRK2 orthologue, LRK-1, in the context of axonal elongation. Epistasis genetic analyses implicated a genetic pathway in which LRK-1 functions downstream of the RAB7L1 orthologue GLO-1, and upstream of the AP-3 complex component APB-3. Further mammalian cell culture studies supported a role for LRRK2 in the regulation of AP-3 complex-dependent intracellular trafficking of proteins bound to the lysosome, such as LAMP1 and LAMP2. In mouse deficiency models, *Rab7l1* KO and *Lrrk2* KO mice displayed strikingly similar phenotypes, with lysosomal inclusions selectively in renal proximal tubule cells. Furthermore, mice with mutations in the *AP3B1* gene (termed *Pearl* mutant mice) have similarly been reported to display prominent age-dependent lysosomal defects in kidney proximal tubule cells, in the absence of CNS pathology^35^ – similar to *Rab7l1* and *Lrrk2* KO mice.

Taken together, these findings implicate a RAB7L1-LRRK2 genetic module in diverse cellular contexts, including axonal termination, endo-lysosomal trafficking, and lysosomal maintenance. Although these module-associated functions appear disparate, our data nonetheless suggest a common downstream mechanism of action, by means of AP-3 complex function. Consistent with this notion, prior studies have shown that AP-3 complex deficiency results in (i) axonal overextension in *C. elegans* neurons^15^, (ii) altered endosomal trafficking of lysosomal membrane proteins (LMPs), such as LAMP1 and LAMP2, in mammalian cells^32^,^33^, and (iii) enlargement of secondary lysosomes in renal proximal tubules of mice^35^,^36^.

The AP-3 complex is required for the endosomal trafficking of a subset of cargo to lysosomes and lysosome-related organelles (LROs), and mutations in AP3B1 subunit are associated with Hermansky-Pudlak Syndrome (HPS), characterized by lysosomal LRO abnormalities leading to albinism, clotting abnormalities, and pulmonary as well as renal dysfunction^32^,^34^. It is thought that the existence of multiple, differentially-expressed isoforms of individual AP-3 components underlies the tissue selectivity of HPS with AP-3 mutations, as well as the varied phenotypes associated with AP-3 component deficiency in mice. For instance, whereas *Pearl* mice with mutations in *AP3B1* gene display prominent lysosomal defects in kidney proximal tubule cells in the absence of CNS pathology^35^, similar to *Rab7l1* and *Lrrk2* KO mice, mice with mutations in *AP3B2* gene – encoding neuron-specific β3B subunit of AP-3 – display neurological and behavioral impairments without renal phenotypes noted^37^. The lack of neurological deficits in typical HPS in patients may reflect redundancy among AP-3 subunit isoforms. We hypothesize that the complex species- and tissue-selective phenotypes associated with genetic mutations in LRRK2 may similarly relate to the existence of redundant evolutionarily-related isoforms, as LRRK2 and its paralog LRRK1 are expressed in a partially overlapping patterns^38^.

The precise molecular mechanism linking RAB7L1, LRRK2 and the AP-3 protein complex still remains unclear. LRRK2 binds to both RAB7L1^10^,^20^ and the AP-3 complex, and the present *C. elegans* genetics findings support the notion that these gene products interact in some way. Recently, it has been reported that LRRK2 physiologically phosphorylates a subset of Rab proteins^39^. Such phosphorylation-dependent regulation of RAB7L1 by LRRK2 is not necessarily contradictory to our genetic model, wherein RAB7L1 acts upstream of LRRK2, as these interacting proteins may regulate each other’s actions. It also remains to be clarified if AP-3 components are regulated directly by LRRK2 kinase activity. Prior studies reported that serine and threonine residues in AP3B1 are highly phosphorylated in cells^40^,^41^,^42^, and we speculated that phosphorylation at those sites may in part be mediated by LRRK2. Furthermore, a proteomic screen identified AP3B1 as a candidate substrate for LRRK2 kinase activity in transfected HEK293 cells^43^. However, our phosphorylation analysis failed to provide evidence for such LRRK2-mediated phosphorylation of AP3B1 *in vivo* or *in vitro* (Supplementary Fig. S5B and data now shown). We thus speculate that LRRK2 may act primarily as a regulatory scaffold for AP-3 complex function.

PD-associated autosomal dominant mutations in LRRK2 have generally been hypothesized to induce pathology through a gain-of-function mechanism, based in part on the observation that a subset of these clinical mutations in LRRK2 lead to increased kinase activity *in vitro* towards generic kinase substrates, as well as to increased LRRK2 autophosphorylation *in vitro* and *in vivo*^16^,^44^,^45^. However, a role for other molecular activities associated with LRRK2, such as GTPase or scaffolding, in the pathogenesis of familial PD has not been excluded. Furthermore, it remains formally possible that a decrease in one or more LRRK2 activities, rather than increased activity, underlie pathology associated with the clinical mutations. We show that the axonal abnormality associated with *lrk-1* deficiency in *C. elegans* is as effectively rescued by wild-type as by PD LRRK2 pathogenic mutant forms (G2019S and R1441C). Similarly, expression of clinical mutant forms of LRRK2 did not significantly alter the cell surface accumulation of LAMPs in mammalian cell culture models, in contrast to LRRK2 deficiency. These data argue against a loss-of-function mechanism for these LRRK2 clinical mutants, but we cannot formally exclude a mechanism by which a modest reduction of LRRK2 functions (below the detection limit of our assays) would play a role in PD pathology *in vivo*. Importantly, disease-related phenotypes in our previous drosophila model overexpressing PD mutant LRRK2 were rescued by RAB7L1 expression^10^; this may support a partial loss-of-function mechanisms for LRRK2 mutants, taken together with our present result that RAB7L1 acts upstream of LRRK2. We also note that α-synuclein, which is accumulated in PD patient brain tissue and is degraded in part through the lysosome compartment, did not appear altered in accumulation in *Lrrk2* or *Rab7l1* deficient kidney in mice at 11–14 months of age (data not shown), consistent with 2 previous studies of *Lrrk2* KO mice^22^,^23^. Thus, α-synuclein does not appear to play a role in the renal pathology observed in these mutant mice at 11–14 month of age (although a role at later time points has been proposed^21^).

Finally, although pre-clinical efforts targeting LRRK2 have focused on the generation of LRRK2 kinase inhibitors, our data support the notion that such compounds may lead to detrimental effects. Indeed, toxicity studies using LRRK2 inhibitors have revealed pathological changes in lung, including lysosomal changes in type II pneumocytes^25^, as is typical of AP-3 complex dysfunction^46^. A detailed understanding of the RAB7L1-LRRK2 pathway may enable the generation of more selective therapeutics for LRRK2-associated PD.

## Methods

### *C. elegans* methods

Nematodes were handled by standard methods^47^. Bristol N2 is used as the wild-type strain. *lrk-1(km17)* mutant and human LRRK2 transgenic lines (P*snb-1*::hLRRK2-WT, G20129S) were described previously^48^. *wyEx3884* (P*lrk-1*::*lrk-1*) and *lrk-1(tm1898)* were provided by Dr. Shen (Stanford University). *glo-1(zu391), muIs32* (P*mec-7*::*gfp*), *fsn-1(gk429)*, *apb-3(ok429)* and *eri-1(mg366)* were obtained from Caenorhabditis genetics center (University of Minnesota, St Paul, MN). The presence of *lrk-1(km17)* allele and LRRK2 transgenes were detected by PCR as described previously^30^,^48^. *muIs32* allele was detected by the fluorescence of GFP, and *glo-1* mutant was recognized by its lack of gut granules^14^. All mutant strains were outcrossed with wild-type N2 for at least four times. Analysis of GFP in live worms was carried out by anesthetizing animals with 50 mM sodium azide on a 4% agarose pad. For observation of ALM axon morphology, worms were examined using Olympus BX51 fluorescence microscope equipped with DP72 digital camera, and the presence of overextension of ALM axon was determined by binary evaluation mode, in which an axon was counted as having an overextension if the end of axon was bent at the tip of nose. For analysis of subcellular localization of CFP::RAB-7 in worm neurons, live animals were anesthetized as above and examined using a Zeiss LSM510 Meta confocal microscope.

### Generation of transgenic *C. elegans*

P*snb-1*::*glo-1* plasmid construct was generated by inserting *glo-1* genomic DNA sequence, which was PCR-amplified from wild-type N2 cDNA, into the SalI-BglII site of the plasmid P*snb-1*::2xNLS-TagRFP (a gift from Dr. Hobert, Columbia University). The plasmid was injected into the gonads of young-adult N2 together with the marker plasmid P*ttx-3*::mCherry and the carrier DNA pBluescript (total concentration: 120 μg/ml) as described^49^. The animals that harbor transgenes as an extrachromosomal array were isolated on the basis of the fluorescence of mCherry.

### DNA constructs

The expression plasmid encoding full-length human LRRK2 cDNA cloned in the p3xFLAG-CMV-10 vector (Sigma-Aldrich) was previously generated^50^. Human LRRK2 deletion constructs were generated from full-length construct by PCR-based mutagenesis. Human AP3B1 cDNA was purchased from Thermo Scientific (clone ID: 3914400), PCR-amplified and cloned into pENTR-D-TOPO vector (Invitrogen). The pENTR construct was transferred to pEF-DEST51 vector (Invitrogen) by recombination using the LR clonase (Invitrogen) to add V5-His-tag at C-terminus. The constructs generated from PCR products were verified by DNA sequencing.

### Cell culture, transfection and RNA interference

Human neuroblastoma SH-SY5Y cell line was maintained in a 1:1 mixture of F12 and Dulbecco’s modified Eagle’s Medium (DMEM) supplemented with 10% fatal bovine serum and penicillin/streptomycin at 37 °C in a 5% CO_2_ atmosphere. Human embryonic kidney (HEK) 293 cell line and mouse neuroblastoma Neuro-2a (N2a) cell line were maintained in DMEM with the same supplements at 37 °C in a 5% CO_2_ atmosphere. Transfection was performed with Lipofectamine 2000 or Lipofectamine 3000 reagent (Invitrogen) according to the manufacturer’s instructions. For RNA interference (RNAi), siRNA oligos (siGENOME SMARTpool, Dharmacon) were transfected into 20–30% confluent cells using LipofectamineRNAiMAX (Invitrogen) at a concentration of 15 nM, and the cells were analyzed 72 hrs after transfection.

### Immunocytochemistry

N2a cells cultured on coverslips were fixed with 4% paraformaldehyde in PBS for 15 min, and then blocked and permeabilized with PBS containing 0.1% TritonX-100 and 3% bovine serum albumin for 30 min. Coverslips were then incubated for 3 hrs with rabbit anti-EEA1 antibody (Cell Signaling Technology) or with mouse anti-LBPA antibody (6C4, Echelon). After washing with PBS, coverslips were incubated for 1 hr with Alexa Fluor 546 goat anti-mouse or rabbit IgG (Invitrogen). Nuclear staining was performed with DRAQ5 (BioStatus). After washing with PBS, coverslips were mounted on a slide glass with PermaFluor Aqueous Mounting Medium (Thermo Scientific). Images were collected with a confocal microscope (SP5, Leica) equipped with Leica LAS AF software. Fluorescent images were cropped, processed and analyzed for assessment of colocalization using ImageJ software (NIH).

### Cell surface biotinylation assay

Cell surface proteins were biotin-labeled and isolated by using the Pierce Cell surface Protein Isolation Kit (Thermo Scientific) according to the manufacturer’s protocol. Briefly, cells grown on a 60 mm culture dish were washed with PBS, and then incubated with EZ-Link Sulfo-NHS-SS-Biotin for 30 min at 4 °C followed by the addition of a quenching solution. Cells were then lysed with 500 μl of lysis buffer containing cOmplete protease inhibitor cocktail (Roche), sonicated, and incubated for 30 min at 4 °C. An aliquot of the lysate was saved (Input fraction). The lysate was mixed with Streptavidin sepharose (GE Healthcare) overnight followed by pull-down and washing. Biotinylated proteins were eluted in 50 μl of the sample buffer containing DTT, and subjected to Western blotting.

### Immunoprecipitation and immunoblotting

For immunoprecipitation, SH-SY5Y cells transfected for 48 hours were lysed with Tris-buffered saline containing 0.5% Triton-X, 1 mM EDTA and protease inhibitor cocktail (Sigma-Aldrich). The lysates were rotated at 4 °C for 1hr followed by centrifugation at 20,000 g for 5 min. The supernatant was added to 30 μl (slurry volume) of Dynabeads protein G (Invitrogen) preincubated without (preclear) and with indicated antibodies and the mixture was rotated for 30 min at 4 °C. The precipitated complexes were washed three times with ice-cold PBS and then eluted with SDS sample buffer for 10 min at 90 °C. SDS-PAGE and Western Blotting were performed with NuPage Bis-Tris Mini Gel and Xcell II Blot Module (Invitrogen) according to manufacturer’s protocols. Antibodies used include: anti-FLAG M2 (Sigma-Aldrich, 1:1000), anti-V5 (Invitrogen, 1:1000), anti-Actin (C4, Abcam, 1:2000), anti-LRRK2 (MJFF2, Epitomics, 1:1000) and appropriate HRP-conjugated secondary antibodies (Jackson ImmunoResearch, 1:2000). Blots were visualized using Supersignal luminol substrate (Thermo Scientific #34075).

### Mouse methods

All procedures with mice were approved by the Institutional Animal Care and Use Committee of the Columbia University Medical Center; all experiments were performed in accordance with the relevant approved guidelines and regulations. *Rab7l1* KO mice were obtained from Wellcome Trust Sanger Institute. *Lrrk2* KO mice were generated previously^18^ and were purchased from The Jackson Laboratories. PCR genotyping of mice using DNA extracted from mouse tails was performed by standard methods with the following primers: 5′-CCA GGC AGT AAC AAG GGG AG-3′, 5′-GAT GTC CAA AAC CCT GTC TGC-3′ for *Rab7l1* wild-type allele, 5′-CCA GGC AGT AAC AAG GGG AG-3′, 5′-TCG TGG TAT CGT TAT GCG CC-3′ for *Rab7l1* mutant allele, 5′-CTC TGA GAG CAG GAG CCG T-3′, 5′-TGC-CTT CCT GGA CAT TAT TCA GCC-3′ for *Lrrk2* wild-type and mutant allele. For histological analyses, mice were perfused and fixed in 4% paraformaldehyde followed by post-fixation at 4 °C overnight. Kidney and brain tissues were subjected to light microscopy or electron microscopy, as described in Supplemental Experimental Procedures. Littermate single or double heterozygous animals were used for the control.

### Quantitative real-time RT-PCR

Total RNA was extracted from cerebral cortex of mouse brain using miRNeasy Mini Kit (Qiagen), and cDNA synthesis was performed from 1 μg of total RNA using the SuperScript III reverse transcriptase (Invitrogen). qPCR reaction mix were set up with the Brilliant II SYBR Green QPCR Master Mix (Agilent Technologies) and the reactions were run using the Mx3000P QPCR System (Agilent Technologies). Primer sequences for real-time PCR are as follows: 5′-CAG CCG AGA TCA CCT GTT TA-3′ and 5′-TCC CAC GGT GGA CTT GTA GT-3′ for Exon 1 of mouse *Rab7l1*, 5′-ATG TTT GAC GTC ACC AAT GC-3′ and 5′-CTT GTT GGC CAA GAG CAG-3′ for Exon 3 of mouse *Rab7l1*, 5′-GGT CTC CTC TGA CTT CAA CA-3′ and 5′-GTG AGG GTC TCT CTC TTC CT-3′ for mouse *Gapdh*. Expression level was normalized by the level of *Gapdh*.

### Histology of mice

For light microscopy, kidney tissues were embedded in paraffin, and 2 μm-thick paraffin sections were stained using Jones methenamine silver with eosin counterstain and with periodic acid-Schiff (PAS) stain. For Oil red O staining, 50 μm sections were generated using a vibratome, and the free-floating sections were stained. For immunostaining of kidney, frozen sections were used. Fresh kidney tissue was removed from mice before fixation, embedded in OCT compound (Tissue-Tek) and immediately frozen in dry ice and stored in a −80 °C deep freezer. Eight μm-thick fresh frozen sections were prepared on glass slides using a Cryostat. Sections were first fixed in PBS containing 1% PFA at 4 °C for 30 min. Endogenous peroxidase activity was inactivated by the treatment with 0.3% H_2_O_2_ for 15 min. Sections were blocked with PBS containing 3% normal goat serum and 0.2% TritonX-100 for 30 min, and then incubated with the primary antibody solution overnight at 4 °C. The antibodies used were anti-LAMP2 (Sigma-Aldrich, 1:500) and anti-Cathepsin B (Millipore, 1:200). Sections were then incubated with biotin-conjugated secondary antibodies (Vector Laboratories) at room temperature for 1 hr followed by reaction with VECTASTAIN Elite ABC Kit (Vector Laboratories). Mouse brain analyses were performed according to our previous papers^51^,^52^. The stained sections mounted on the glass slides were examined using Olympus BX51 microscope.

### Electron microscopy

Kidney cortex was cut into 1 mm cubes, fixed in 2.5% glutaraldehyde in cacodylate buffer, post-fixed in 1% buffered aqueous osmium tetroxide, dehydrated in graded ethanols, transferred to propylene oxide, and embedded in epoxy resin. After trimming of the plastic blocks, ultrathin (silver) sections were cut on an ultramicrotome using a diamond knife and mounted on copper grids, followed by staining with uranyl acetate and lead citrate. Electron microscopic images were photographed using a JEOL 1011 electron microscope equipped with Gatan digital imaging system.

### Protein preparation from mouse tissues

Preparation of the tissue protein was according to the conventional method. Tissues were taken out from mouse brains (midbrain or striatum) or kidneys, and homogenized in RIPA Buffer (#89900, Thermo Scientific) containing protease inhibitor cocktails (P8340, Sigma-Aldrich) and phosphatase inhibitor cocktails (#78420, Thermo Scientific) on ice. The homogenates were centrifuged at 14,000 rpm for 30 min at 4 °C after 30-min incubation on ice. The supernatants were collected and subjected to conventional SDS-PAGE and Western Blotting analyses. SuperSep Phos-tag gels were used for the detection of phospho-AP3B1 (Wako Pure Chemical Industries). The antibodies used here were: Actin (ab3280, Abcam), AP3B1 (ARP33647_P050, Aviva Systems Biology), LIMP2/SCARB2 (PA5-20540, Thermo Scientific), and ZnT3 (#197003, Synaptic Systems).

### Statistical analysis

All numerical results are obtained from a minimum of four independent experiments unless otherwise stated, and the data are represented as mean ± SEM. Statistical significance of difference between two groups was calculated by the two-tailed unpaired Student’s t-test of the means. For multiple-means comparisons, we calculated statistical significance of difference by one-way analysis of variance (ANOVA) with Bonferroni correction or with Fisher’s PLSD post hoc test.

## Additional Information

**How to cite this article**: Kuwahara, T. *et al*. LRRK2 and RAB7L1 coordinately regulate axonal morphology and lysosome integrity in diverse cellular contexts. *Sci. Rep.* **6**, 29945; doi: 10.1038/srep29945 (2016).

## Supplementary Material

Supplementary Information
