# Supplementary material for: LRRK2 and RAB7L1 coordinately regulate axonal morphology and lysosome integrity in diverse cellular contexts
Source: Sci Rep. 2016 Jul 18;6:29945. doi: 10.1038/srep29945 (PMC4947924; doi:10.1038/srep29945)
Supplement: Supplementary Information [file srep29945-s1.pdf]

Supplementary information for

LRRK2 and RAB7L1 coordinately regulate axonal morphology and lysosome integrity in diverse cellular contexts

Authors: Tomoki Kuwahara[1,2], Keiichi Inoue[1], Vivette D. D'Agati[3], Tetta Fujimoto[2], Tomoya Eguchi[2], Shamol Saha[4], Benjamin Wolozin[4], Takeshi Iwatsubo[2], and Asa Abeliovich[1,]*

Affiliations:

[1]Departments of Pathology, Cell Biology and Neurology, and Taub Institute, Columbia University, New York, NY, 10032, USA.

[2]Department of Neuropathology, Graduate School of Medicine, The University of Tokyo, Tokyo, 113-0033, Japan.

[3]Department of Pathology and Cell Biology, College of Physicians and Surgeons, Columbia University, New York, NY, 10032, USA.

[4]Department of Pharmacology and Experimental Therapeutics and Department of Neurology, Boston University School of Medicine, Boston, MA, 02118, USA.

*To whom correspondence should be addressed. E-mail: aa900@columbia.edu

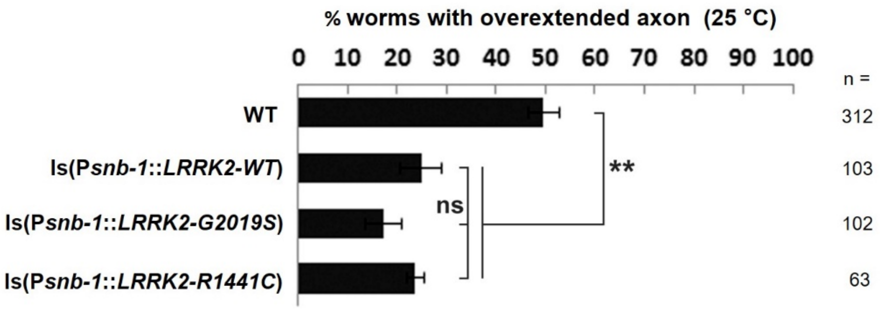

Supplementary Figure S1. The effect of PD-associated LRRK2 mutations on ALM axon termination (related to Figure 1)

Overextension of ALM axons at 25°C was significantly suppressed by transgenic expression of either human LRRK2 wild-type (WT), LRRK2-G2019S or LRRK2-R1441C, and the effects were comparable among these strains. Data represent mean ± SEM, **: $p<0.01$; Sample numbers n are listed to the right of bar graphs.

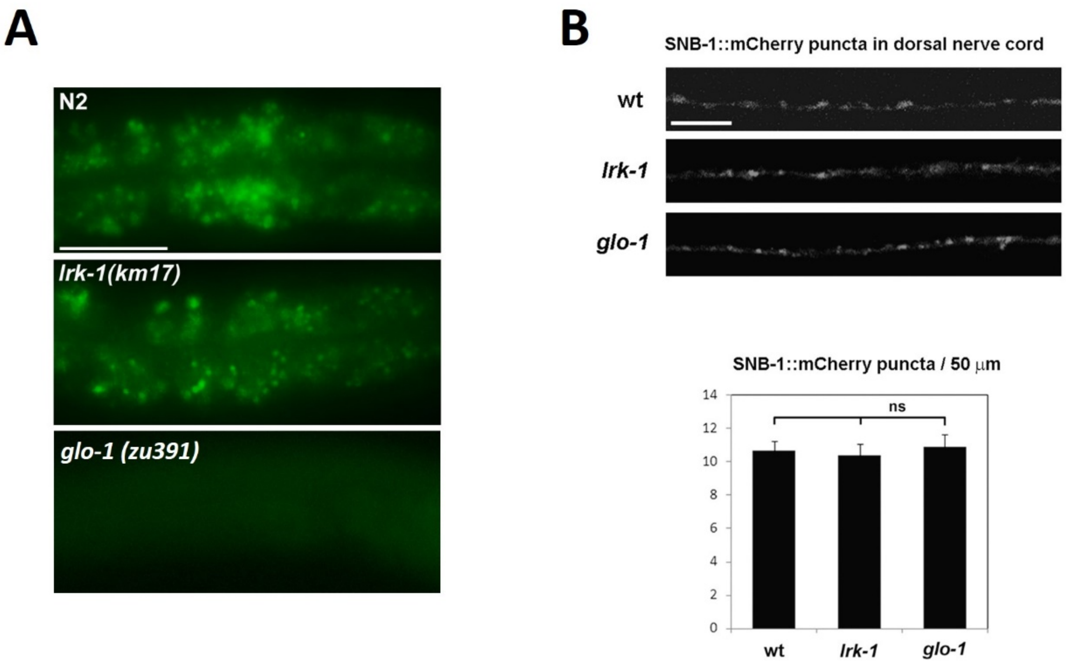

Supplementary Figure S2. The role of *C. elegans* LRK-1 and GLO-1 in endo-lysosomal system (related to Figure 2)

(A) Fluorescence of gut granules in intestinal cells of wild-type N2, *lrk-1(km17)* and *glo-1(zu391)* mutants. Unlike *glo-1* mutants, *lrk-1* mutants did not show reduction in gut granules, as quantified by fluorescence microscopy of birefringent puncta. Scale bar = 50 μm. **(B)** Top: fluorescence of the presynaptic marker SNB-1::mCherry puncta along the dorsal nerve cord of the indicated mutant strains harboring *hpIs78* (P*unc-25*::*snb-1*::*mCherry*). Scale bar = 10 μm. Bottom: quantitative analysis of the number of SNB-1::mCherry puncta in 50 μm region along the dorsal nerve cord of the indicated strains. No significant difference was observed among wild-type, *lrk-1,* and *glo-1* mutants. Data represent mean ± SEM, n = 15 per group; ns: not significant.

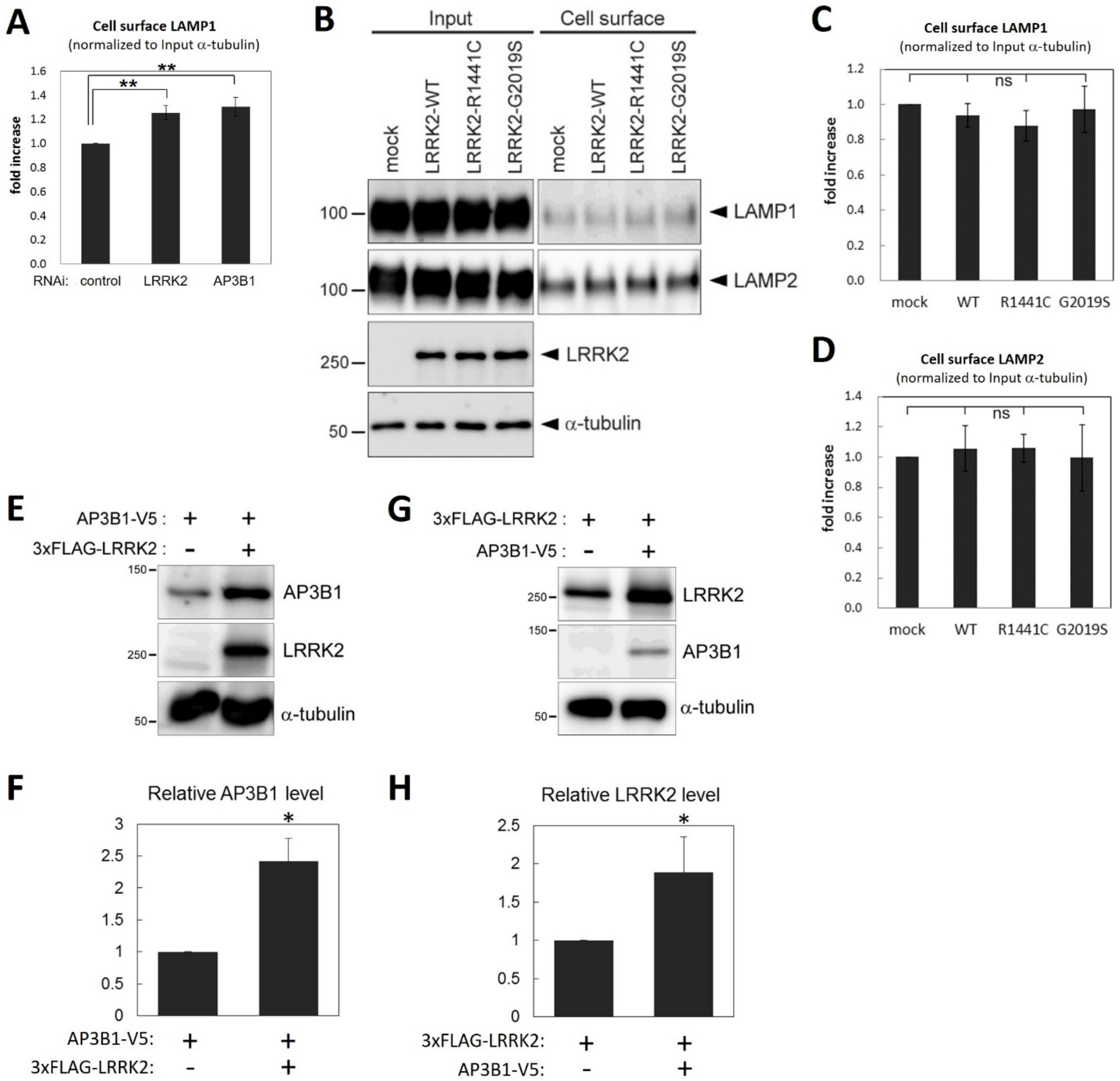

Supplementary Figure S3. Analyses of LRRK2-AP3B1 relationships in mammalian cells (related to Figure 3)

(A) Quantification of cell surface accumulation of LAMP1 as shown in Fig. 3A from four independent experiments. Signals were normalized by the intensities of α-tubulin bands in input fractions. Data represent mean ± SEM, **: p<0.01. **(B-D)** Cell-surface biotinylation analysis of HEK293 cells transfected with the vector encoding 3xFLAG-tagged LRRK2-WT, LRRK2-R1441C, LRRK2-G2019S or

empty vector (mock) as indicated. Representative western blot images (B) and the quantification of cell surface accumulation of LAMP1 (C) or LAMP2 (D) from four independent experiments are shown. Signals were normalized by the intensities of α-tubulin bands in input fractions. Data represent mean ± SEM, ns: not significant. **(E-H)** Western blot analysis of the lysates from HEK293 cells overexpressing V5-tagged AP3B1 only, 3xFLAG-tagged LRRK2 only, or both proteins as indicated. The relative expression levels of AP3B1 (E, F) or LRRK2 (G, H) were assessed by measuring the band intensity of each protein and normalized by that of α-tubulin as a loading control. The representative images (E, G) and the quantification of the band intensities from four independent experiments (F, H) are shown. Data represent mean ± SEM, *: $p<0.01$.

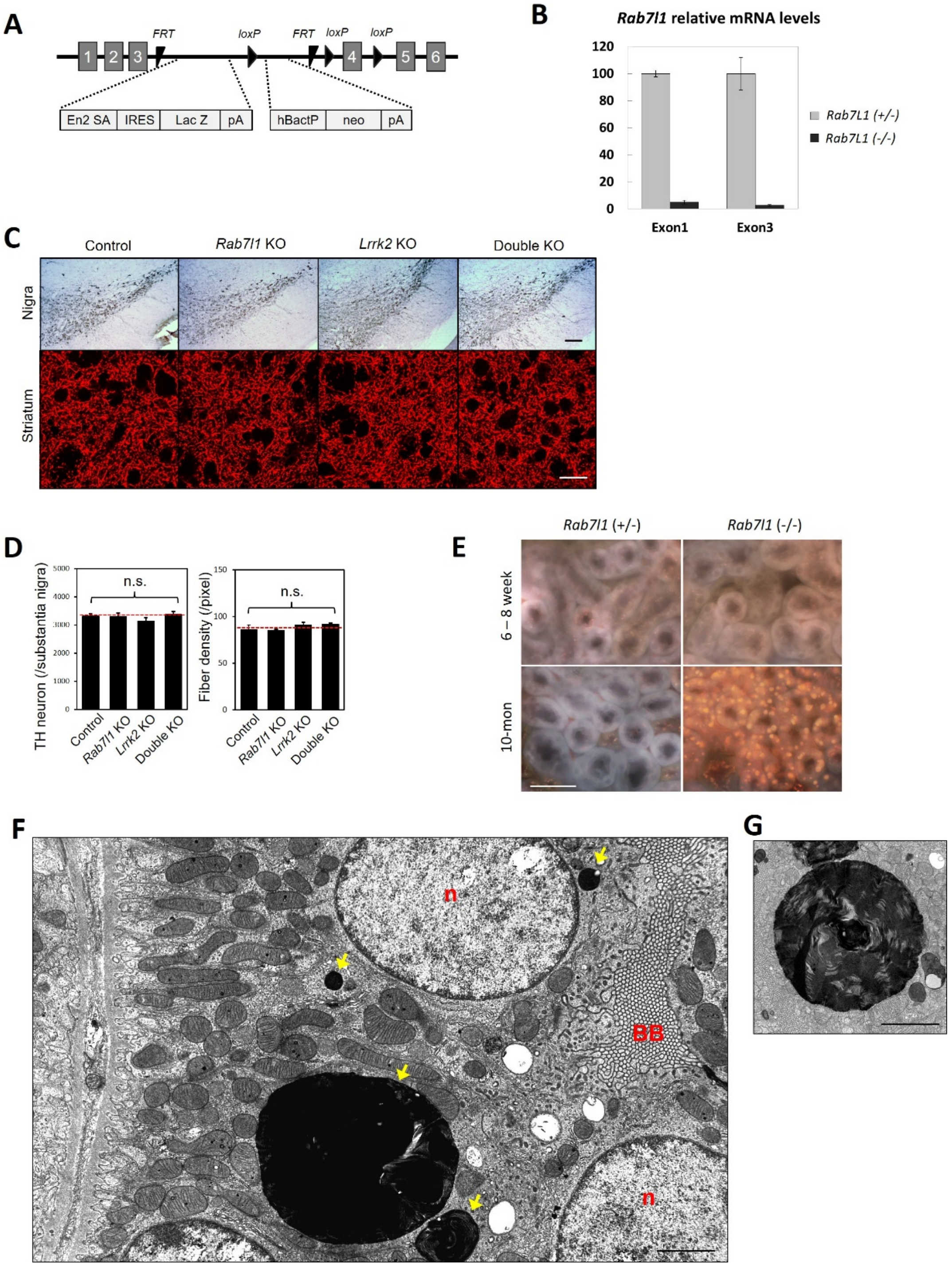

Supplementary Figure S4. Histochemistry of *Rab7l1* and *Lrrk2* deficient mice (related to Figure 4)

(A) A schematic diagram showing the genomic structure of *Rab7l1* KO mice. The allele contains large insertions flanked by two FRT sites between exon3 and exon4 of the *Rab7l1* gene. **(B)** Quantitative RT-

PCR analysis of *Rab7l1* mRNA levels in the cerebral cortex of control (heterozygous, *Rab7l1*(+/-)) and *Rab7l1* KO (homozygous, *Rab7l1*(-/-)) mice. mRNA levels in both exon 1 and exon 3 regions of *Rab7l1* gene were analyzed, and the levels in *Rab7l1*(+/-) mice were normalized to 100. Expression of *Rab7l1* gene was mostly diminished in *Rab7l1* KO mice. Data represent mean ± SEM, n = 4. **(C, D)** Unaltered CNS dopaminergic systems in 11- to 14-month-old *Rab7l1*, *Lrrk2*, or double KO mice. Panels in C show the representative pictures of the nigral and striatal sections immunostained with anti-tyrosine hydroxylase (TH) antibody. There were no significant differences in the number of TH-positive neurons of the substantia nigra (D, left) or in TH-positive axonal densities of the striatum (D, right) among all indicated mutant mice. Data represent mean ± SEM. n =12 slices from 3 animals per genotype; n.s.: not significant. Scale bars in C = 250 μm (nigra) and 20 μm (striatum). **(E)** Auto-fluorescent granules in kidney proximal tubule cells were not apparent in younger (6-8-week-old) *Rab7l1* KO mice, unlike those in older (10-month-old) mice. Scale bar = 50 μm. **(F, G)** Electron microscopic analysis revealed the accumulation of electron-dense material (arrowhead) within enlarged secondary lysosomes in proximal tubule cells of 10-month-old *Rab7l1* KO mice. The inclusion often have a whorled or lamellated texture (G). Original magnification: ·5,000. Scale bars = 2 μm.

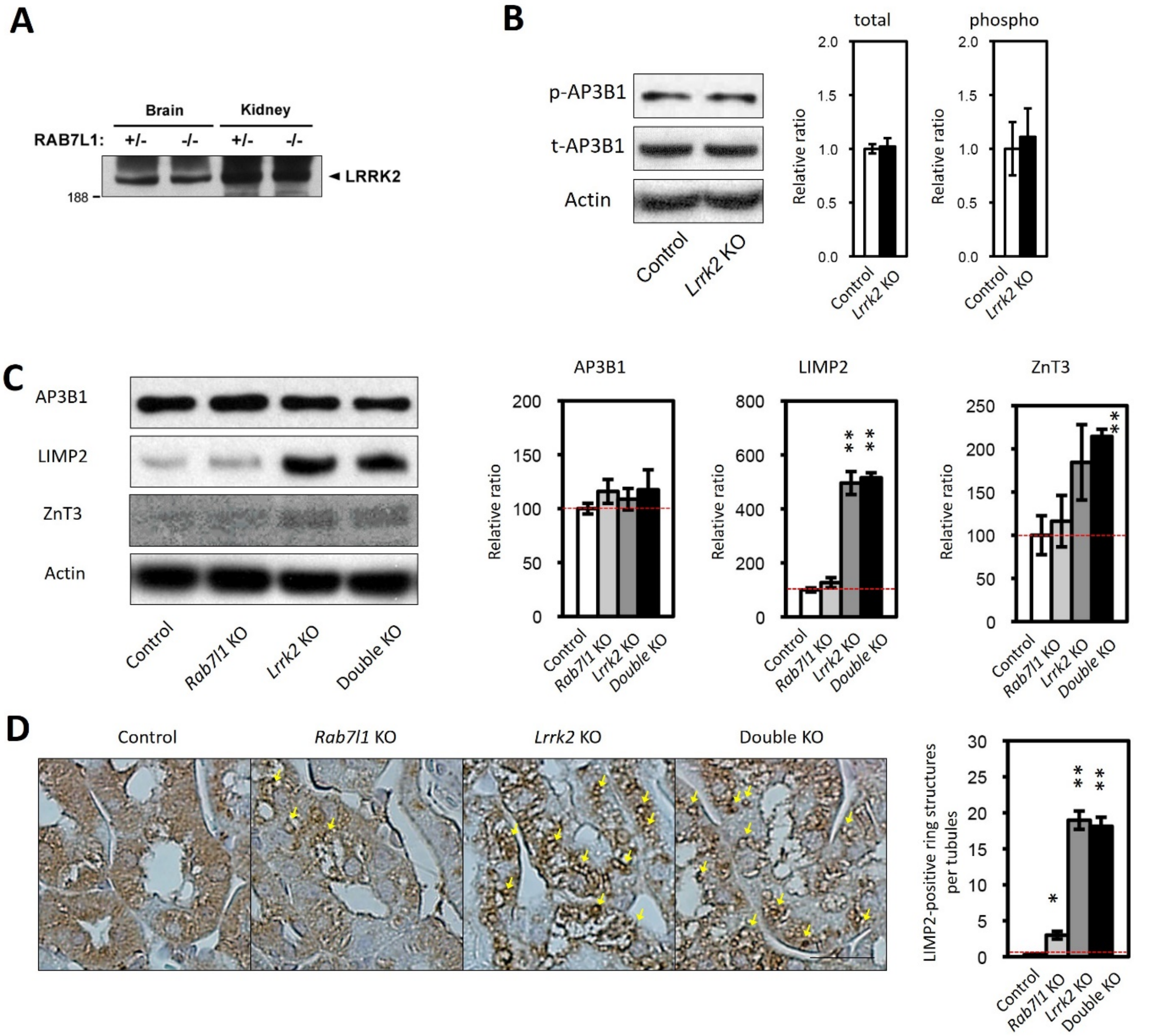

Supplementary Figure S5. Molecular analysis of lysosome-related proteins, including AP-3 and its cargo proteins, in mutant mouse tissues (related to Figure 4)

(A) Representative immunoblot for LRRK2 in the brain and kidney lysates from control and *Rab7l1* KO mice at 2 months of age. RAB7L1 deficiency did not affect the level of LRRK2. **(B)** No alterations in total (t-) and phosphorylated (p-) AP3B1 proteins in 11- to 14-month-old *Lrrk2* KO kidney tissues. Signals were normalized by Actin for total AP3B1 and by total AP3B1 for phospho-AP3B1. Data represent mean ± SEM. n = 5 per genotype. **(C)** The AP-3 cargo proteins, LIMP2 and ZnT3, were significantly accumulated in kidney extracts from 10-month-old *Lrrk2* KO or double KO mice. (left)

Representative images of immunoblot analyses of AP3B1, LIMP2, ZnT3, and Actin. (right) Quantification of immunoblot analyses. Signals were normalized by Actin. n = 4 per genotype. **<0.01. **(D)** IHC analysis of kidney sections from 10-month-old mice with an antibody to LIMP2. Left panels: positively-stained circular structures (arrows) were abundant in proximal tubule cells of *Lrrk2* KO kidney sections and in double KO sections; such structures were apparent, but to a lesser extent, in *Rab7l1* KO kidney sections, whereas only diffuse staining was seen in kidney sections from control animals. Scale bar = 20 μm. Right panel: quantification of LIMP2 -positive circular structures in kidney sections of mouse strains (as indicated), presented per sectioned tubule structure. *, p<0.05; **, p<0.01.
